# Co-metabolic Effect of Glucose on Methane Production and Phenanthrene Removal in an Enriched Phenanthrene-Degrading Consortium Under Methanogenesis

**DOI:** 10.3389/fmicb.2021.749967

**Published:** 2021-10-12

**Authors:** Ziyan Zhou, Yanqin Wang, Mingxia Wang, Zhifeng Zhou

**Affiliations:** College of Resources and Environment, Southwest University, Chongqing, China

**Keywords:** anaerobic digestion, methanogenesis, phenanthrene degradation, methane production, co-metabolism

## Abstract

Anaerobic digestion is used to treat diverse waste classes, and polycyclic aromatic hydrocarbons (PAHs) are a class of refractory compounds that common in wastes treated using anaerobic digestion. In this study, a microbial consortium with the ability to degrade phenanthrene under methanogenesis was enriched from paddy soil to investigate the cometabolic effect of glucose on methane (CH_4_) production and phenanthrene (a representative PAH) degradation under methanogenic conditions. The addition of glucose enhanced the CH_4_ production rate (from 0.37 to 2.25mg⋅L^−1^⋅d^−1^) but had no influence on the degradation rate of phenanthrene. Moreover, glucose addition significantly decreased the microbial α-diversity (from 2.59 to 1.30) of the enriched consortium but showed no significant effect on the microbial community (*R*^2^=0.39, *p*=0.10), archaeal community (*R*^2^=0.48, *p*=0.10), or functional profile (*R*^2^=0.48, *p*=0.10). The relative abundance of genes involved in the degradation of aromatic compounds showed a decreasing tendency with the addition of glucose, whereas that of genes related to CH_4_ synthesis was not affected. Additionally, the abundance of genes related to the acetate pathway was the highest among the four types of CH_4_ synthesis pathways detected in the enriched consortium, which averagely accounted for 48.24% of the total CH_4_ synthesis pathway, indicating that the acetate pathway is dominant in this phenanthrene-degrading system during methanogenesis. Our results reveal that achieving an ideal effect is diffcult *via* co-metabolism in a single-stage digestion system of PAH under methanogenesis; thus, other anaerobic systems with higher PAH removal efficiency should be combined with methanogenic digestion, assembling a multistage pattern to enhance the PAH removal rate and CH_4_ production in anaerobic digestion.

## Introduction

Anaerobic digestion is a promising strategy for the disposal of diverse categories of waste materials, such as domestic wastewater, agricultural waste, food industry waste, and municipal solid waste, because it has the advantages of low energy consumption, less investment in land and management, nutrient reclamation, and biogas generation (Gallert and Winter, 1997; He et al., 2005; Stabnikova et al., 2008; Chan et al., 2009; Holm-Nielsen et al., 2009; Lytras et al., 2021). Methane (CH_4_) is the principal component of biogas produced by anaerobic digestion and can be used as clean, renewable bioenergy that can substitute fossil fuels (Holm-Nielsen et al., 2009; Haberl et al., 2012; Awe et al., 2017; Li et al., 2019). Additionally, many kinds of organic hazards, such as polycyclic aromatic hydrocarbons (PAHs), BTEX, chloramphenicol, polyacrylamide, and antibiotics, can be effectively removed from the initial substrates and converted into CH_4_ *via* anaerobic digestion (Kunapuli et al., 2007; Agarry and Owabor, 2011; Hu et al., 2018; Guo et al., 2019; Bianco et al., 2020). Therefore, how to efficiently harness the anaerobic digestion process for the improvement of CH_4_ production and removal the rate of organic pollutants has long been a global challenge.

Co-metabolism is a traditional approach adopted to enhance the degradation of refractory pollutants and CH_4_ production in the anaerobic digestion system, which can vigorously promote the growth and activity of indigenous microbes by supplying some popular carbon sources (e.g., glucose, fructose, and sucrose) for microbes to the target system (Li et al., 2016a,b; Xie et al., 2016; Khan et al., 2017; Hadibarata et al., 2018; Feng et al., 2019). Glucose is one of the most favorable carbon (energy) sources for heterotrophic microbes and has been frequently used as a co-metabolic substrate to improve CH_4_ production and accelerate the degradation of refractory pollutants in diverse environments (Ambrosoli et al., 2005; Jing et al., 2017; Khan et al., 2017; Shu et al., 2021; Zhou et al., 2021). For example, the addition of glucose was found to significantly enhance the degradation of fluorene, phenanthrene, and pyrene under an anaerobic denitrification system (Ambrosoli et al., 2005), and the addition of glucose effectively alleviated the inhibitory effect of gallic acid on CH_4_ production in an anaerobic digestion system (Mousa and Forster, 1999). Therefore, it seems that glucose can be used as a prioritized metabolic substrate to efficiently remove refractory pollutants in anaerobic digestion systems (Li et al., 2016b; Khan et al., 2017).

Polycyclic aromatic hydrocarbons are a group of persistent organic pollutants with intensive toxicity and wide distribution, posing a serious threat to environmental and human health (Xue and Warshawsky, 2005; Couling et al., 2010; Zhou et al., 2018). Previous investigations have reported that co-metabolism triggered by glucose could significantly accelerate the degradation of PAHs under both aerobic and anaerobic digestion environments (Ambrosoli et al., 2005; Ma et al., 2011; Qin et al., 2018; Tripathi et al., 2020; Al Farraj et al., 2021). For example, the addition of glucose was reported to significantly enhance the degradation of PAHs (benzopyrene, anthracene, and pyrene) when treated with some pure bacterial species, such as *Cellulosimicrobium*, *Sphingomonas*, and *Bacillus* (Khanna et al., 2012; Qin et al., 2018; Al Farraj et al., 2021). Additionally, PAH degradation under methanogenesis is thermodynamically feasible and the degradation of PAHs can be coupled with methanogenesis, suggesting that remediation of PAHs pollution *via* the anaerobic digestion system should be a potentially ideal method marked by the properties of energy conservation and resource reuse (Chang et al., 2005; Dolfing et al., 2009; Berdugo-Clavijo et al., 2012; Wan et al., 2012; Nzila, 2018; Ye et al., 2019; Wang et al., 2021b). Whether methanogens can directly participate in the degradation of PAHs remains unclear, but some potential keystone microbes in this process have been identified. In addition to methanogenic archaea *Methanosaeta*, *Methanoculleus*, and *Methanosarcina*, some syntrophic bacteria, such as *Methylibium*, *Legionella*, *Clostridiaceae*, *Citrobacter*, and *Pseudomonas*, have also been found essential for PAH degradation and CH_4_ generation (Berdugo-Clavijo et al., 2012; Zhang et al., 2012). Thus, in anaerobic digestion, methanogenesis is the pivotal step that not only can harness biogas (CH_4_) production but also can be coupled with PAH degradation. However, the co-metabolic effect induced by glucose on PAH degradation and CH_4_ production under methanogenesis has seldom been reported.

In this study, we enriched the phenanthrene-degrading microbial consortium under methanogenic conditions from a long-term rice paddy, which was used to investigate the co-metabolic effect of glucose on CH_4_ production and phenanthrene degradation during methanogenesis. The response of the microbial community and functional composition to the bio-stimulation of glucose in the enriched microbial consortium was further analyzed based on the results of metagenomic sequencing. This study aimed to examine whether the co-metabolic substrate (glucose) could enhance CH_4_ production and phenanthrene degradation under the methanogenesis system and explore the community and functional responses of the phenanthrene-degrading microbial consortium to the bio-stimulation of glucose, providing useful information for the enhancement of CH_4_ production and PAHs elimination during the anaerobic digestion of waste containing PAHs.

## Materials and Methods

### Enrichment of Methanogenesis Consortium of Phenanthrene Degradation

**Figure 1:**
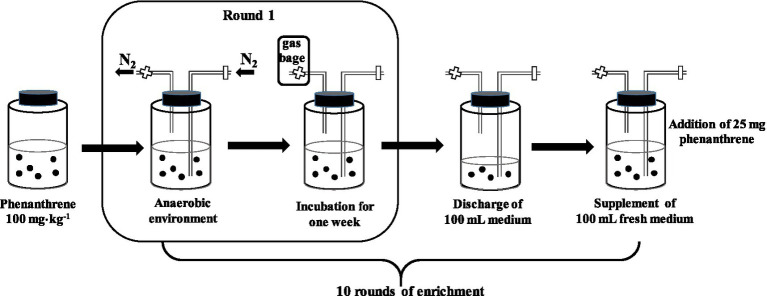
The diagrammatic sketch showing the enrichment procedure of the phenanthrene-degrading consortium under methanogenesis.

In this experiment, the methanogenesis consortium was enriched from a paddy soil sample collected from a long-term (approximately 30years) rice paddy field (N29°48', E106°48') in the Beibei District (Chongqing, China) in July 2020. A brief enrichment procedure is shown in Figure 1 and described as follows.

Four types of nutrition solutions, selective media for methanogens (solution A), inorganic salt solution (solution B), trace element solution (solution C), and vitamin solution (solution D) were prepared for the enrichment of the target microbial consortium, the ingredients of which are listed in Supplementary Table 1 (Ferguson and Mah, 1983; Brauer et al., 2006). First, 200g of fresh paddy and 200ml of sterilized and distilled water were added into a borosilicate glass bottle (1L) pre-added with 50mg phenanthrene, and then 100ml of mixed solution containing A, B, C, and D (A:B:C:D=20:1:0.2:0.2) was added into the bottle with a final phenanthrene concentration of 100mg⋅kg^−1^; the bottle was sealed using a butl rubber stopper that was pierced through by two glass tubes, one of which was inserted into the liquid and the other was above the liquid surface (Figure 1). The pure nitrogen gas (N_2_) was filled into the bottle *via* the gas inlet for 10min to expel the air *via* the outlet (Figure 1); after the inlet was closed and a gas collection bag was connected to the outlet, the bottle was shaken (100r⋅min^−1^) and incubated in the dark at 30°C for 1week for the first round of enrichment. Subsequently, 100ml of supernatant in the bottle was discharged and replaced by 100ml of fresh mixed solution (containing 25mg phenanthrene), and then the second round of enrichment was conducted *via* the same procedure as that for the first round. Using this method, six bottles of the target microbial consortium were obtained, each of which had undergone 10 rounds of enrichment (10weeks).

### Experiment Design to Examine the Effect of Glucose on Methane Production and Phenanthrene Degradation

**Figure 2:**
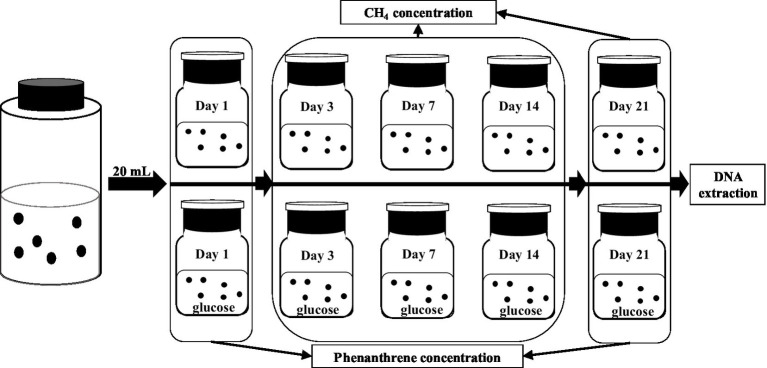
The diagram showing the experiment design to investigate the cometabolic effect of glucoe on the MPR and PDR of the enriched consortium.

The experimental design is illustrated in Figure 2. The six bottles of consortium obtained were equally and randomly divided into two groups, denoted as PME (without glucose) and PMEG (with glucose), respectively. In a serum bottle (100ml) with 0.25mg phenanthrene, 20ml suspension from one bottle of the enriched consortium, 30ml mixed medium, and 6ml sterilized and distilled water was added for the PME treatment. For the PMEG treatment, sterilized and distilled water was substituted with glucose solution (0.1mol⋅L^−1^). After sealing with butyl rubber and an aluminum cap, the bottle was filled with N_2_ for 10min to generate an anaerobic environment, and then shaken (100 r⋅min^−1^) and incubated in the dark at 30°C for 21days. The CH_4_ concentration in the bottle was measured on days 3, 7, 14, and 21, and the phenanthrene concentration was detected on days 1 and 21. After incubation, genomic DNA was extracted from each microbial consortium for metagenomic sequencing.

### Determinations of Methane Concentration and Phenanthrene Content

On days 3, 7, 14, and 21 of the incubation period, the CH_4_ concentration in the headspace of each serum bottle was sampled and then detected using a gas chromatograph (Agilent Technologies 7890A, United States) equipped with a flame ionization detector (FID) under the following conditions – carrier gas, N_2_; flow rate, 30cm^3^⋅min^−1^; oven temperature, 50°C; FID temperature, 200°C (standardized by Wang and Wang, 2003) – and the methane production rate (MPR, mg CH_4_⋅L^−1^⋅d^−1^) was calculated according to the method previously described by Zhang et al. (2011).

The phenanthrene concentration of each culture sample in the serum bottle was detected on days 1 and 21 during the incubation, and the brief detection procedure was as follows. The culture in each serum bottle was shaken well and then transferred into a separating funnel (250ml) with 1.5g NaCl. Subsequently, the bottle was rinsed with 50ml of n-hexane, also transferred into the funnel. After shaking for 5min, the funnel was statically placed to stratify the organic and aqueous (containing soil particles) phases. Next, the organic phase was retained, while the residual phase was re-extracted using n-hexane. After mixing and then clarifying using anhydrous sodium sulfate, the extracted organic phase was concentrated using a rotary evaporator. According to the method described by Zhou et al. (2017b), the solvent (n-hexane) was replaced with methanol, and then the phenanthrene content was detected using a gradient elution method (mobile phase: methanol and water; flow rate: 0.6ml⋅min^−1^; column temperatuer: 30°C; detection wavelength: 250nm) by an HPLC (Hitachi High-Technologies Corporation, Japan). Finally, the phenanthrene degradation rate (PDR, %) was determined using the following equation:


$$\mathrm{phenanthrene degradation rate}\;\left(\%\right)=\frac{C_{1}-C_{21}}{C_{1}}\times 100$$

where *C*_1_ and *C*_21_ are the phenanthrene content (mg⋅L^−1^) of the culture in the serum bottle on days 1 and 21, respectively.

### DNA Extraction and Metagenomic Sequencing of the PME and PMEG Samples

Total genomic DNA was extracted from 0.5ml culture in each serum bottle (thoroughly shaken for a sufficient homogeneity) using MoBio UltraClean™ soil DNA isolation kits (San Diego, CA, United States) according to the manufacturer’s instructions, and the integrity and purity of DNA were checked with 1% agarose gel electrophoresis and NanoDrop200 spectrophotometer (Thermo, United States), respectively. The extracted DNA was sent to Majorbio Bio-Pharm Technology Co., Ltd. (Shanghai, China) for metagenomic sequencing, and the brief procedure is described as follows.

The extracted DNA was fragmented to an average length of approximately 400bp using Covaris M220 (Gene Company Limited, China) for the construction of paired-end (PE) library, and then NEXTflex™ Rapid DNA-Seq (Bioo Scientific, United States) was used to construct the PE library. After the ligation of specific DNA adapters with the blunt end of fragments, PE sequencing was performed on the Illumina NovaSeq platform (Illumina Inc., San Diego, CA, United States) using NovaSeq Reagent Kits according to the manufacturer’s standard procedure. Using fastp (version 0.20.0)^1^ on the free online cloud platform of Majorbio,^2^ the adaptor sequences and low-quality reads (length below 50bp, minimum quality lower than 20, and with N bases) in the raw reads of metagenomic sequencing were removed and trimmed. These pair-end and single-end reads with high quality were then assembled into contigs using MEGAHIF (version 1.1.2)^3^ based on succinct de Bruijn graphs. These assembled contigs being or longer than 300bp were preserved as the final assembly result. Open reading frames (ORFs) in these available contigs were identified using MetaGene (Noguchi et al., 2006), and the identified ORFs equal to or longer than 100bp were retrieved and translated into amino acid sequences using the NCBI translation table. The predicted contigs were clustered using CD-HIT (version 4.6.1)^4^ with 90% sequence identity and 90% coverage, and the longest gene sequence in each gene cluster was isolated as the representative for the construction of a non-redundant gene set. The high-quality reads in each sample were mapped to the non-redundant gene set under 95% identity using SOAPaligner (version 2.21),^5^ and the gene abundance in the sample was calculated. The functional annotation of the representative sequences in the non-redundant gene set was performed using Diamond (version 0.8.35)^6^ against the Kyoto Encyclopedia of Genes and Genomes (KEGG) database (version 94.2)^7^ with an e-value cutoff of 1e^−5^.

### Data Analysis

The independent t-test in the SPSS 11.5 (SPSS, Chicago, United States) was used to check the difference of the parameters (MPR, PDR, and relative abundance of functional genes) between the PME and PMEG treatments, and *p* values below 0.05 were considered as significant. In R software with the Vegan package, the principal coordinate analysis (PCoA) and Adonis test (checking the difference in significance of the composition in the PME and PMEG treatments) were performed, and the Mental test was conducted with the command of “envit” to calculate the correlations between the composition (microbial and functional) and MPR or PDR. Variance partition analysis (VPART) was applied to quantify the explained variance of MPR and PDR for the microbial or functional composition, conducted using the Vegan package with the command of “rdavp.” All figures were generated using the Ggplot2 package in the R software.

## Results

### Effect of Glucose on MPR and PDR of the Phenanthrene-Degrading Consortium Under Methanogenesis

**Figure 3:**
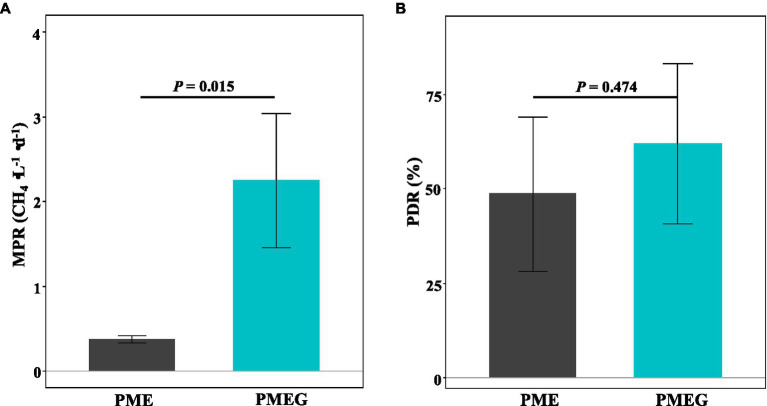
The MPR **(A)** and PDR **(B)** in the enriched consortium treatments of without (PME) and with (PMEG) additon of glucose. *Error bars* indicate SDs (*n*=3). The *p* values above the horizontal lines are the results of independent *t*-test.

The MPR and PDR in the no-glucose (PME) and glucose-added (PMEG) treatments are shown in Figure 3. The MPR of the PME (0.37mg⋅L^−1^⋅d^−1^) treatment was significantly lower (*p*<0.05) than that of the PMEG (2.25mg⋅L^−1^⋅d^−1^) treatment (Figure 3A). Although the value of PDR of PME (48.69%) was lower than that of PMEG (62.10%), no significant difference was detected in the PDR between these two treatments (Figure 3B). This result indicated that the addition of glucose promoted the activity of methanogens in the enriched microbial consortium but did not affect the degradation of phenanthrene.

### Whole Microbial Community and Archaeal Community in the PME and PMEG Treatments

**Figure 4:**
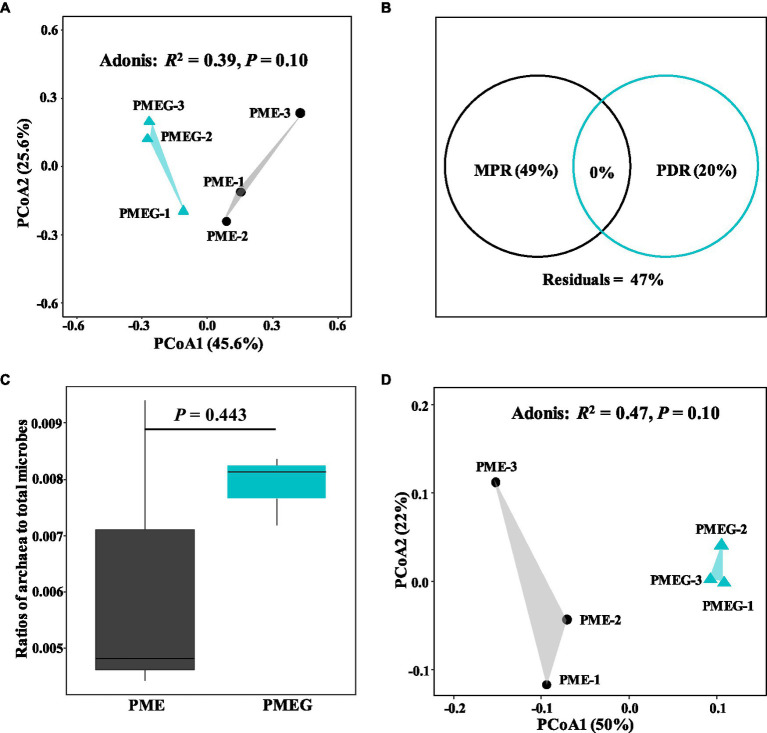
The principal coordinate analysis (PCoA) profile based on the microbial species composition in the PME and PMEG treatments **(A)** and variance partitioning analysis showing the portions explained by MPR and PDR for the microbial community structure **(B)**; the ratios of archaea to total microbes in the PME and PMEG treatments **(C)** and the PCoA profile based on the archaeal species composition in the PME and PMEG treatments **(D)**.

**Figure 5:**
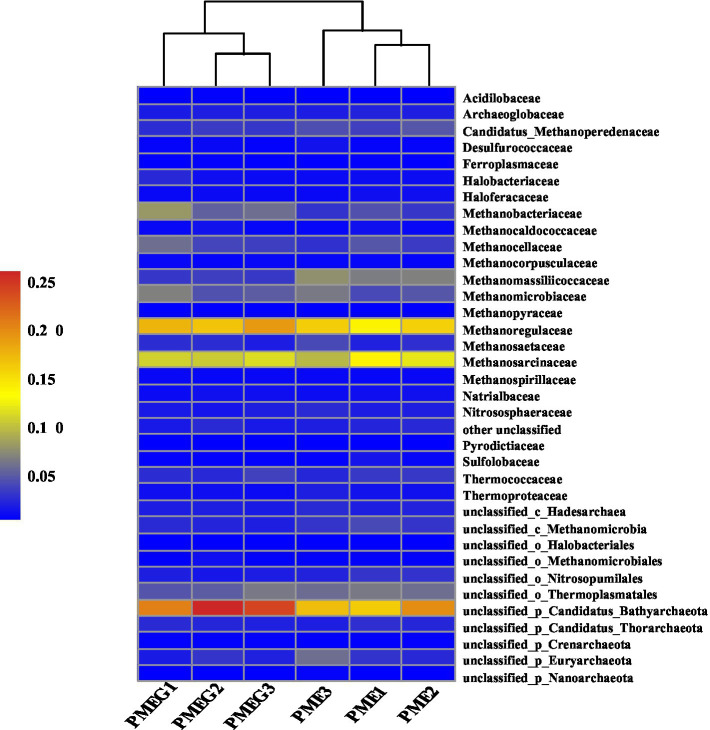
Heatmap showing the archaeal community composition based on the level of family in the PME and PMEG treatments.

The result of microbial α-diversity (Supplementary Figure 1) indicates that the PME treatment had a significantly higher (*p*<0.05) α-diversity than the PMEG treatment. The PCoA profile (Figure 4A) constructed from the microbial species composition based on the metagenomic sequencing indicated that the points of the PME and PMEG treatments were in a narrow area (axis1, −0.27–0.43; axis2, −0.22–0.23). Moreover, the Adonis test revealed no significant difference (*R*^2^=0.39, *p*=0.10) between the microbial community structures of the PME and PMEG treatments (Figure 4A). However, the Mantel test indicated that MPR (*r*^2^=0.91, *p*=0.025) and PDR (*r*^2^=0.92, *p*=0.028) were significantly correlated with the microbial community structure in the enriched consortium (Table 1), which could explain 49 and 20% of the variance in the community structure, respectively (Figure 4B). The heatmap generated from the archaeal family composition in each treatment is shown in Figure 5 and indicates that all treatments had a similar composition of archaeal family with Methanoregulaceae and Methanosarcinaceae as the dominant classified families. Furthermore, our results showed that the ratio of archaea to total microbes in the PME was lower than that in the PMEG, but no significant difference was detected between them (Figure 4C). Additionally, no significant difference was detected in the archaeal community structure (based on the level of species) between the PME and PMEG treatments (Figure 4D), and only MPR had a significant relationship (*r*^2^=0.91, *p*=0.034) with the archaeal community structure of the enriched consortia (Table 1).

**Table 1:** Mantel correlations among microbial community, archaeal community, functioanl composition, methane production rate (MPR), and phenanthrene degradation rate (PDR) in the enriched consortium.

Items	MPR		PDR	
	*r* ^2^	*p* value	*r* ^2^	*p* value
Microbial community	0.91	0.025	0.92	0.028
Archaeal community	0.91	0.034	0.84	0.067
Functional composition	0.90	0.026	0.88	0.049

### PCoA Analysis of a Functional Profile Based on Metagenomic Sequencing in the PME and PMEG Treatments

**Figure 6:**
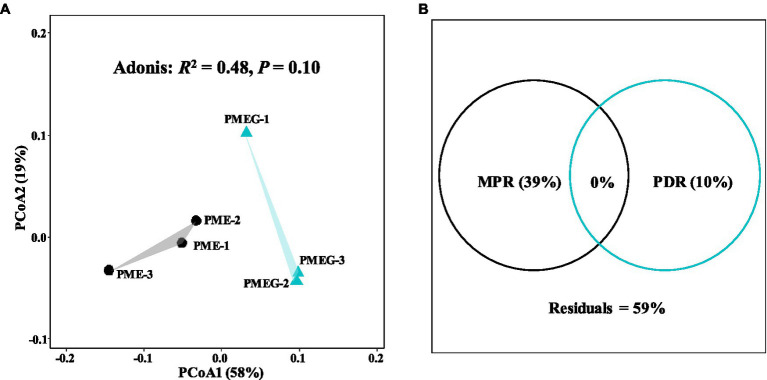
The PCoA profile based on the functional enzymes composition in the PME and PMEG treatments **(A)** and variance partitioning analysis showing the portions explained by MPR and PDR for functional enzymes composition **(B)**.

No significant difference was detected in the macro-functional composition based on the KEGG annotation between the PME and PMEG treatments (Supplementary Figure 2; Supplementary Table 2). Thus, we further analyzed the composition of functional genes in these two treatments by using PCoA analysis; the results are shown in Figure 6A. Similar to the PCoA profile (Figure 4A) based on the microbial composition, the sample points of the PME and PMEG treatments were distributed in a narrow area (axis1, −0.14-0.99; axis2, −0.03-0.10), and the Adonis test showed no significant difference (*R*^2^=0.48, *p*=0.10) in the functional composition between these two treatments (Figure 6A). MPR and PDR were also significantly correlated with the functional components in the enriched microbial consortium (Table 1), explaining 39 and 10% of the variance in the functional composition, respectively (Figure 6B).

### Relative Abundance of Genes Related to Aromatic Compounds Degradation and CH_4_ Synthesis in the PME and PMEG Treatments

**Figure 7:**
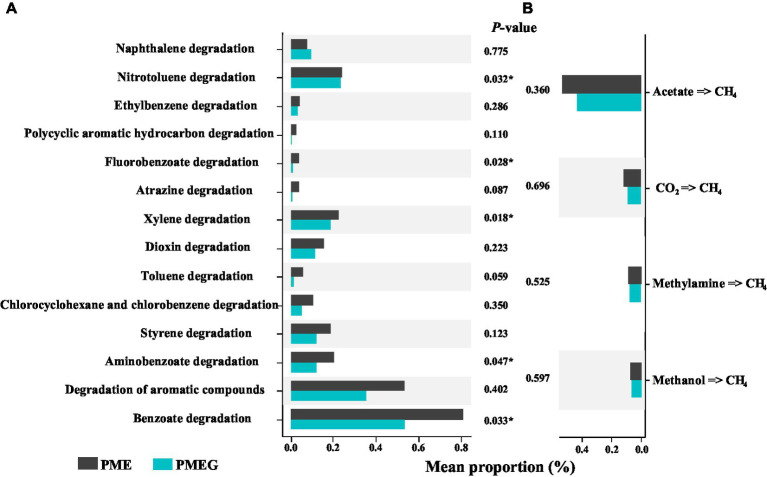
Comparision of the relative abundances of genes involved in the degradation of aromatic commpounds **(A)** and methane synthesis **(B)** between the PME and PMEG treatments.

To investigate the potential mechanism by which glucose affects the MPR and PDR in the enriched microbial consortium, we further compared the relative abundance of genes related to the degradation of aromatic compounds and CH_4_ synthesis in the PME and PMEG treatments (Figure 7). Fourteen types of genes responsible for the degradation of aromatic compounds were detected based on the KEGG pond of metagenomic sequencing, 13 types of which showed higher values of relative abundance in the PME treatment than in the PMEG treatment (Figure 7A). The results of the independent *t*-test indicated that the relative abundance of genes associated with nitrotoluene (PME: 0.243%, PMEG: 0.235%), fluorobenzoate (PME: 0.039%, PMEG: 0.009%), xylene (PME: 0.226%, PMEG: 0.189%), aminobenzoate (PME: 0.206%, PMEG: 0.121%), and benzoate (PME: 0.809%, PMEG:0.537%) degradation in the PME treatment were significantly higher than those in the PMEG treatment (Figure 7A). For the genes involved in CH_4_ synthesis, genes related to 4 types of CH_4_ synthesis pathways – acetate (dominant in all treatments with an average proportion of 48.20%), CO_2_, methylamine, and methanol pathways – were detected based on the results of metagenomic sequencing, and no significant difference was observed between these two treatments (Figure 7B).

## Discussion

In this study, the CH_4_ production rate of the enriched microbial consortium was significantly improved by the addition of glucose (Figure 3A), which was consistent with findings in the literature that glucose could enhance CH_4_ production in anaerobic digestion systems (Bucha et al., 2018; Maletic et al., 2018; Vu and Min, 2019; Li et al., 2021). Compared with phenanthrene, glucose can be more quickly and easily utilized and subsequently converted into carbon dioxide (CO_2_), hydrogen (H_2_), and acetate by the microbes in the enriched consortium under anaerobic conditions, supplying feasible substrates for methanogens to synthesize CH_4_ (Khan et al., 2017; Zhang et al., 2019). However, our results indicated that the addition of glucose did not promote the degradation of phenanthrene (Figure 3B). Several studies have reported the enhancement of PAH degradation caused by glucose addition or other co-metabolic substrates under anaerobic conditions (Yuan and Chang, 2007; Ma et al., 2011; Li et al., 2016a). For example, Li et al. (2016a) reported that glucose addition enhanced the degradation rate of petroleum hydrocarbons by 200%, while the promotion of petroleum hydrocarbons degradation was mainly attributed to the stimulation of the biocurrent triggered by glucose. Maletic et al. (2018) found that glucose addition improved CH_4_ production but had no effect on the degradation of PAHs, which is consistent with our current results and should be explained by the following reasons. The utilization of phenanthrene was delayed by the addition of glucose, which should start after the complete consumption of glucose, and then the metabolism of glucose resulted in the improvement of CH_4_ production. However, the glucose addition might neither promote microbial activity to degrade phenanthrene nor induce the microbial consortium to produce phenanthrene-degrading enzymes during methanogenesis.

This study revealed that the addition of glucose decreased the α-diversity of the enriched consortium (Supplementary Figure 1), which is consistent with reports that glucose addition decreased the microbial α-diversity in some enriched consortia (Li et al., 2016a; Goldford et al., 2018; Zhang et al., 2019; Shu et al., 2021), indicating the selectivity of a single carbon source on the microbial community. Additionally, glucose addition had no significant influence on the microbial community of the enriched phenanthrene-degrading consortium (Figure 4A). The addition of co-metabolic substrates has usually been shown to trigger shifts in the microbial community structure in anaerobic digestion systems (Li et al., 2016a,b; Zhang et al., 2019). In these studies, the wastes treated in their systems contained inartificial microbes originating from soil or wastewater, which should be complex, unstable, and fictile, and then susceptible to external carbon sources such as glucose. By contrast, the microbial consortium in our study had undergone an enrichment period of 10 rounds supplied with the sole carbon source (phenanthrene) and nutrient components, which should be a stable ecosystem insensitive to the external addition of glucose. Sundh et al. (2003) also reported that glucose addition significantly enhanced CH_4_ yield, but only slightly affected the microbial community in an anaerobic digestion system fed with a synthetic substrate. Furthermore, our result showed that MPR and PDR were significantly correlated with the microbial community composition of the enriched consortium (Table 1). Generally accepted is that the microbial community structure determines its function (de Souza and Santos, 2018; Zhou et al., 2019). The close linkage between CH_4_ production and microbial communities in diverse ecosystems has been confirmed (Shrestha et al., 2011; Edwards et al., 2015; Wang et al., 2020; Watanabe et al., 2020). However, the strong selectivity of PAHs to environmental microbes has led to a strong relationship between PAH degradation and microbial community structure (Zhou et al., 2017a; Spini et al., 2018; Wang et al., 2021a). Moreover, our results revealed that MPR explained a greater percentage of the variance in the microbial community than PDR (Figure 4B), indicating that the former might be more dependent on the microbial community structure than the latter, which might be due to the different microbial mechanisms of CH_4_ synthesis and phenanthrene degradation. In detail, the CH_4_ synthesis process might involve a series of steps driven by diverse microbes, including the primary metabolism of carbon sources, the transformation of the intermediates, and the generation of substrates (CO_2_, H_2_, and acetate) for methanogens (Maletic et al., 2018), whereas the degradation rate of phenanthrene might be determined by limited microbes responsible for the cleavage of the benzene ring (Meckenstock and Mouttaki, 2011). Therefore, MPR should be more important in explaining the variance of the microbial community than PDR in the phenanthrene-degrading system during methanogenesis.

In this study, the PCoA profile based on the composition of functional genes indicated that the macro-function of the enriched consortium was not affected by the addition of glucose, which was consistent with the response of the microbial community structure to glucose (Figure 6A; Supplementary Figure 2). Additionally, the significant correlation between the functional composition and MPR or PDR (Table 1), and the result of VPART analysis based on the functional composition (Figure 6B), were similar to the results based on the microbial community structure, further confirming the uniformity between the microbial community and its functions. In this study, the genes involved in the degradation of some refractory compounds and CH_4_ biosynthesis were selected from the functional pool of KEGG for further analysis. The relative abundance of those degrading genes showed a decreasing tendency due to the addition of glucose (Figure 7A), indicating that the metabolic characteristic of the microbial community also followed the principle of “glucose repression.” Glucose (monosaccharide) will hamper the *Escherichia coli* from utilizing lactose (disaccharide), known as “glucose repression,” suggesting that an easy-utilized carbon source will be preferentially metabolized by microbes and thus hinder the utilization of the complex one (Rose et al., 1991). Nevertheless, the relative abundance values of degrading genes in the PME and PMEG treatments were of the same order of magnitude (Figure 7A), which the following reasons might explain. First, the DNA samples were collected at the end of the incubation (day 21) when the glucose had been exhausted; second, the metagenomic sequencing in this study was based on the DNA level, which could not reflect the actual transcription level of those degrading genes. The relative abundance of the genes related to CH_4_ synthesis pathways was not significantly different between the PME and PMEG treatments, and the pathway of CH_4_ synthesis *via* acetate was dominant in this enriched consortium (Figure 7B). The acetate pathway has been reported to be the key CH_4_ synthesis pathway in paddy soils (Conrad, 1999; Glissmann and Conrad, 2002; Masuda et al., 2018). Acetate is also an essential intermediate or terminal product in the degradation of crude oil (a major source of PAHs) or some PAHs (naphthalene, phenanthrene, anthracene, and pyrene) during methanogenesis (Dolfing et al., 2008, 2009; Jones et al., 2008). Therefore, CH_4_ synthesis *via* the acetate pathway should be dominant in the anaerobic phenanthrene digestion system during methanogenesis.

## Conclusion

In this study, we examined the co-metabolic effect of glucose on CH_4_ production and phenanthrene degradation in a phenanthrene-degrading consortium under methanogenesis. We found that glucose addition significantly enhanced the CH_4_ production rate of the enriched consortium but had no influence on its ability to degrade phenanthrene. Glucose addition significantly decreased the α-diversity of the enriched microbial consortium but showed no significant effect on either the microbial community or the macro-function of the enriched consortium. Glucose addition only slightly decreased the relative abundance of the genes involved in the degradation of aromatic compounds, and the acetate pathway was dominant for CH_4_ synthesis in this anaerobic phenanthrene digestion system under methanogenesis. These results indicate that it is difficult to remarkably enhance PAH degradation in a single-stage anaerobic digestion system under methanogenesis *via* the addition of co-metabolic substrates. Therefore, we propose that a multistage and combined pattern comprising methanogenesis and other more efficient anaerobic digestion pathways of PAH (sulfate-reducing or denitrification) should be a promising strategy to enhance the CH_4_ production and PAH degradation rate in the anaerobic digestion of PAH-containing wastes.

## Data Availability Statement

The datasets presented in this study can be found in online repositories. The names of the repository/repositories and accession number(s) can be found at: https://www.ncbi.nlm.nih.gov/, PRJNA744876.

## Author Contributions

ZiZ and YW conducted the experiment, analyzed the data, and wrote the original draft. MW arranged the investigation consortium and contributed to the phenanthrene and methane detection. ZhZ designed the experiment and contributed to the edit, data analysis, and review of the manuscript. All authors contributed to the article and approved the submitted version.

## Funding

This work was supported by the National Natural Science Foundation of China (41771501).

## Conflict of Interest

The authors declare that the research was conducted in the absence of any commercial or financial relationships that could be construed as a potential conflict of interest.

## Publisher’s Note

All claims expressed in this article are solely those of the authors and do not necessarily represent those of their affiliated organizations, or those of the publisher, the editors and the reviewers. Any product that may be evaluated in this article, or claim that may be made by its manufacturer, is not guaranteed or endorsed by the publisher.

## Footnotes

^1^ https://github.com/OpenGene/fastp

^2^ cloud.majorbio.com

^3^ https://github.com/voutcn/megahit

^4^ http://www.bioinformatics.org/cd-hit/

^5^ http://github.com/ShujiaHuang/SOAPaligner

^6^ http://github.com/bbuchfink/diamond

^7^ http://www.genome.jp/kegg/
