# Supplementary material for: Co-metabolic Effect of Glucose on Methane Production and Phenanthrene Removal in an Enriched Phenanthrene-Degrading Consortium Under Methanogenesis
Source: Front Microbiol. 2021 Oct 12;12:749967. doi: 10.3389/fmicb.2021.749967 (PMC8546250; doi:10.3389/fmicb.2021.749967)
Supplement: Supplementary file 1 [file Data_Sheet_1.pdf]

Co-metabolic Effect of Glucose on Methane Production and Phenanthrene Removal in An Enriched Phenanthrene-Degrading Consortium Under Methanogenesis

Ziyan Zhou, Yanqin Wang, Mingxia Wang, Zhifeng Zhou*

College of Resources and Environment, Southwest University, Chongqing, Beibei, China

*** Correspondence:**

Zhifeng Zhou

zhouzhf@swu.edu.cn

Supplementary material:

Supplementary Table 1 Ingredients of mediums used to enrich methanogens in this study

Supplementary Table 2 Sequence numbers of genes involved in different functional categories on KEEG levels 1and 2 in the PME and PMEG treatments

Supplementary Figure 1. Microbial *Shannon* indices in the PME and PMEG treatments.

Supplementary Figure 2. Comparison of the macro-metabolic pathways (KEGG level 1) between PME and PMEG treatments based on the Wilcoxon test.

Supplementary Table 1 Ingredients of mediums used to enrich methanogens in this study

Mediums	Medium ingredients (1000 mL)
Solution A	NH_4Cl 1.00 g, $MgCl_2$ 1.00 g, K_2HPO_4 0.40 g, KH_2PO_4 0.40 g, cysteine 0.50 g, resazurin (1%) 1 mL
Solution B	K_2HPO_4 6.00 g, KH_2PO_4 6.00 g, $(NH_4)_2SO_4$ 6.00 g, NaCl 12.00 g, $MgSO_4 \cdot 7H_2O$ 2.60 g, $CaCl_2 \cdot 2H_2O$ 0.16 g
Solution C	$MgSO_4 \cdot 7H_2O$ 3.00 g, $MnSO_4 \cdot 2H_2O$ 0.50 g, $FeSO_4 \cdot 7H_2O$ 0.10 g, $CoCl_2 \cdot 6H_2O$ 0.10 g, $CaCl_2 \cdot 2H_2O$ 0.10 g, $CuSO_4 \cdot 5H_2O$ 0.01 g, $ZnSO_4 \cdot 7H_2O$ 0.10 g, $KAl(SO_4)_2$ 0.01 g, $NiCl_2 \cdot 6H_2O$ 0.02 g, $Na_2MoO_4 \cdot 2H_2O$ 0.01 g, NaCl 1.0 g, H_3BO_3 0.01 g
Solution D	biotin 2.00 mg, folacin 2.00 mg, thiamine 5.00 mg, riboflavin 5.00 mg, pyridoxine 10.00 mg, cobalamin 0.10 mg, niacin 5.00 mg, calcium pantothenate 5.00 mg, protogen 5.00 mg, para-aminobenzoic acid 5.00 mg

Supplementary Table 2 Sequence numbers of genes involved in different functional categories on KEEG levels 1and 2 in PME and PMEG treatments

KEGG Categories		Treatments					
**Level 1**	**Level 2**	**PME-1**	**PME-2**	**PME-3**	**PMEG-1**	**PMEG-2**	**PMEG-3**
Cellular Processes	Cell growth and death	329418	402526	398878	338950	335162	312394
Cellular Processes	Cell motility	548928	809240	732370	771494	758988	753850
Cellular Processes	Cellular community - eukaryotes	190	346	486	846	474	960
Cellular Processes	Cellular community - prokaryotes	1109962	1679200	1816324	1167426	1305830	1248714
Cellular Processes	Transport and catabolism	186396	168064	185760	105150	107744	104502
Environmental Information Processing	Membrane transport	1699286	2231642	2222666	2025162	2153348	2035440
Environmental Information Processing	Signal transduction	1281758	1741334	1717620	1419594	1472876	1398296
Environmental Information Processing	Signaling molecules and interaction	890	1764	430	1288	460	1548
Genetic Information Processing	Folding, sorting and degradation	588902	770760	721562	706802	625428	636442
Genetic Information Processing	Replication and repair	843304	1108178	922586	1058706	1022950	1052410
Genetic Information Processing	Transcription	94948	126474	104244	114596	117092	117698
Genetic Information Processing	Translation	906514	1161778	1007942	1145896	1112786	1095822
Human Diseases	Cancers: Overview	157290	190910	184764	142030	116640	132594
Human Diseases	Cancers: Specific types	29510	36170	46268	17940	11608	13958
Human Diseases	Cardiovascular diseases	50598	68130	81194	47672	47052	51998
Human Diseases	Drug resistance: Antimicrobial	365808	534806	425760	468458	501980	503920
Human Diseases	Drug resistance: Antineoplastic	106472	135606	127816	107492	87996	103636
Human Diseases	Endocrine and metabolic diseases	116506	164396	128128	126660	125796	130428
Human Diseases	Immune diseases	16482	14210	12172	15386	12474	13814
Human Diseases	Infectious diseases: Bacterial	183104	236364	253290	220334	189810	186910
Human Diseases	Infectious diseases: Parasitic	15576	16826	18442	7200	12512	18610
Human Diseases	Infectious diseases: Viral	4670	5152	8096	2514	2218	3062
Human Diseases	Neurodegenerative diseases	58034	70534	76860	48948	51140	47776

Human Diseases	Substance dependence	770	1166	2930	1460	952	2480
Metabolism	Amino acid metabolism	3026282	3512634	3475462	3269124	2829280	2859190
Metabolism	Biosynthesis of other secondary metabolites	455458	548524	458194	497064	475330	510404
Metabolism	Carbohydrate metabolism	3042410	3683476	3235626	3707764	3268420	3196660
Metabolism	Energy metabolism	2161334	2737880	2657608	2441014	2103470	2050672
Metabolism	Global and overview maps	3330496	3965272	3849824	3713946	3233686	3277072
Metabolism	Glycan biosynthesis and metabolism	535022	664092	552860	593128	588268	577902
Metabolism	Lipid metabolism	888012	948694	903484	907964	728576	687596
Metabolism	Metabolism of cofactors and vitamins	1811066	2196752	2160510	2171640	1677132	1683510
Metabolism	Metabolism of other amino acids	728966	852178	791638	751328	668186	670476
Metabolism	Metabolism of terpenoids and polyketides	489376	552136	487880	493798	417826	418448
Metabolism	Nucleotide metabolism	1299344	1652552	1524942	1563416	1446868	1481366
Metabolism	Xenobiotics biodegradation and metabolism	740370	745742	859046	656406	510536	491628
Organismal Systems	Aging	121850	159584	161978	138162	111362	110526
Organismal Systems	Circulatory system	4216	13456	14200	2452	2250	2220
Organismal Systems	Development	2672	102	104	164	120	120
Organismal Systems	Digestive system	11464	14010	14276	8890	15646	33348
Organismal Systems	Endocrine system	238918	273102	269666	182446	168638	171594
Organismal Systems	Environmental adaptation	49874	57930	91722	60006	51098	49264
Organismal Systems	Excretory system	14724	15560	15380	9146	9488	7696
Organismal Systems	Immune system	43980	54146	76672	51506	46720	48170
Organismal Systems	Nervous system	40300	51054	55618	41888	38124	52554
Organismal Systems	Sensory system	26	34	144	90	90	98

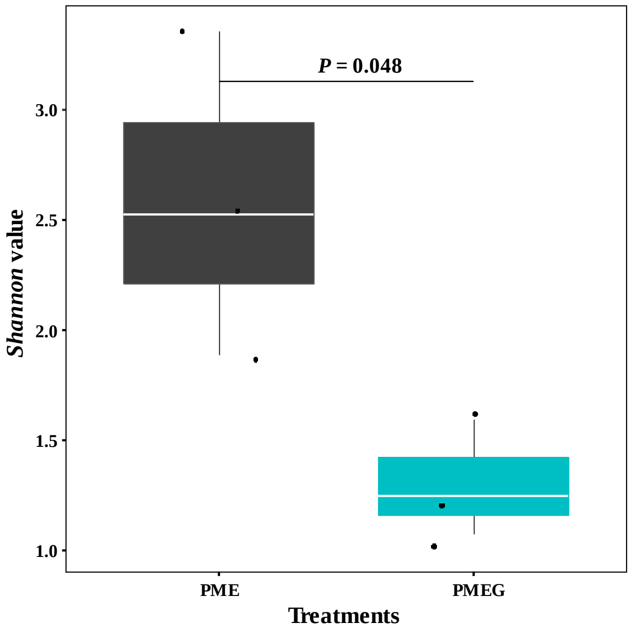

Supplementary Figure 1. Microbial *Shannon* indices in the PME and PMEG treatments.

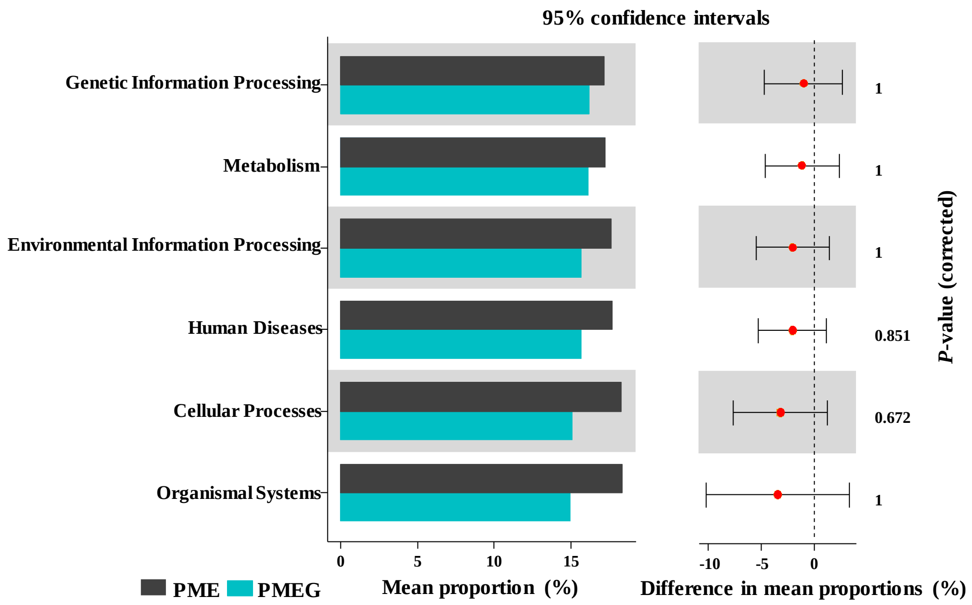

Supplementary Figure 2. Comparison of the macro-metabolic pathways (KEGG level 1) between PME and PMEG treatments based on the Wilcoxon test.
